# ASO Author Reflections: Examining the Outcomes of Adding Immunotherapy to Adjuvant Chemoradiation in Pathologic Stage II–IIIB Non-Small Cell Lung Cancer

**DOI:** 10.1245/s10434-025-17879-5

**Published:** 2025-07-22

**Authors:** Natasha Venugopal, Jorge Raul Vazquez-Urrutia, Junjia Zhu, Asato Hashinokuchi, Shinkichi Takamori, Takefumi Komiya

**Affiliations:** 1 Department of Medicine, Penn State Health Milton S. Hershey Medical Center, Hershey, PA USA; 2 Department of Public Health Sciences, Penn State College of Medicine, Hershey, PA USA; 3 Department of Surgery and Science, Graduate School of Medical Sciences, Kyushu University, Fukuoka, Japan; 4 Department of Surgery and Science, Graduate School of Medical Sciences, Kyushu University, Fukuoka, Japan; 5 Division of Hematology Oncology, Penn State College of Medicine, Hershey, PA USA

**Keywords:** Non-small cell lung cancer, Adjuvant chemotherapy, Radiation, Immunotherapy, Atezolizumab

## Past

Stage II–IIIB non-small cell lung cancer (NSCLC) carries high recurrence rates after resection, with 3-year recurrence-free survival of 58% and 38%, respectively.^1^ National Comprehensive Cancer Network (NCCN) guidelines recommend chemoradiation (CT+RT) for inoperable cases, given evidence of improved outcomes.^2^ While studies such as IMpower-010, KEYNOTE-091/PEARLS, CheckMate 816, and KEYNOTE-671 support immunotherapy in resectable NSCLC, they excluded adjuvant radiation, leaving a gap in evidence for patients receiving CT+RT.^3,4^ To address this, we evaluated the role of immune checkpoint inhibitors (ICIs) within CT+RT strategies for this high-risk population, using a large dataset. 

## Present

Our study analyzed 8235 patients with pathologic stage II–IIIB NSCLC from the NCDB, where the addition of ICIs to adjuvant CT was associated with increased overall survival (OS), with a 2-year OS rate of 90.1% versus 86.0% without ICIs (hazard ratio [HR] 0.66; *p* < 0.001). However, the addition of an ICI to a CT+RT strategy showed no OS benefit, with 2-year OS rates of 77.8% with ICI and 76.1% without (HR 0.85; *p* > 0.05). These findings persisted after propensity score matching, adjusting for confounding variables, further reinforcing our observations.^5^ Our findings suggest that the addition of ICIs to chemoradiation may not add benefit compared with chemotherapy alone, which raises important considerations for treatment planning in these populations.

## Future

Although the NCDB is a valuable resource, the lack of detailed patient data and limited time frame of only 2 years resulted in limitations in our study. As such, biomarker-stratified analysis and studies with extended follow-up remain important areas for future research. Despite the limitations, our results echo recent negative studies investigating the use of ICIs with CT+RT in unresectable stage III NSCLC (PACIFIC2) and limited-stage small-cell lung cancer (NRG-LU005). Further investigations with prospective studies are indicated to verify these results so that treatment guidelines for patients with NSCLC can be optimized.

## Data Availability

The datasets analyzed during the current study are available via the NCDB upon request.
